# Daily routine versus on-demand chest radiograph policy and practice in adult ICU patients- clinicians’ perspective

**DOI:** 10.1186/s12880-018-0248-6

**Published:** 2018-04-03

**Authors:** Abdullah Al Shahrani, Khaled Al-Surimi

**Affiliations:** 1 King Abdulaziz Medical City, Ministry of National Guard Health Affairs, Riyadh, Saudi Arabia; 2 Department of Health Systems and Quality Management, College of Public Health and Health Informatics, King Saud bin Abdulaziz University for Health Sciences, Riyadh, Saudi Arabia; 3 Primary Care and Public Health Department, School of Public health, Imperial College London, London, UK

**Keywords:** Portable chest radiograph, Intensive care unit, ICU clinicians, On-demand CXR, Daily routine (CXR) Saudi Arabia

## Abstract

**Background:**

Chest radiographs are taken daily as a part of routine investigations in Intensive care unit (ICU) patients. They are less effective and unlikely to alter the management of the majority of these patients compared to the radiographs obtained when indicated. According to the American College of Radiology (ACR) Appropriateness criteria, only selective ordering of chest radiographs is recommended, including elderly or high risk patients. The aim of this study was to identify and assess the clinician’s perspective in abandoning the current practice of daily routine chest radiograph and replacing with the on-demand radiograph in Saudi hospitals.

**Methods:**

This was a cross-sectional study. A valid self-administered questionnaire was distributed to all clinical staff members working in ICUs in the major tertiary hospitals in Saudi Arabia. The study population was primarily the ICU intensivists (physicians), nurses and respiratory therapists (RT). The data collected were statistically processed using SPSS version 20.0; descriptive and inferential analyses were done.

**Results:**

Out of 730 questionnaires sent, we received only 495 completed questionnaires with a response rate of 67.8%. Majority of them (*n* = 351) are working at academic hospitals. About half of the respondents (*n* = 247) are working in an open-format ICUs. Findings showed that the daily routine chest X-ray was performed in almost 96.8% of ICUs patients, which the majority of the clinical staff members (73%) thought that this current daily routine CXR protocol in the ICUs should be replaced with the on-demand CXR policy. Interestingly, the differences in demographic and work-related characteristics had no significant impact on the clinician’s view and supported moving to on-demand CXR policy and practice.

**Conclusions:**

The daily routine CXR is still a common practice in most of the Saudi hospitals ICUs although enough empirical evidence shows that it can be avoided. We observed that intensivists support the change of the current practice and recommend an on-demand CXR policy likely to be followed in intensive care management.

**Electronic supplementary material:**

The online version of this article (10.1186/s12880-018-0248-6) contains supplementary material, which is available to authorized users.

## Background

Chest radiography is the most frequently done procedure among intubated and mechanically ventilated patients [1]. There have been two different schools of thought regarding this procedure; the chest radiographs (CXRs) performed routinely without any specific indication (referred to daily routine CXRs) and another strategy is the use of CXRs with specific reason (referred to on-demand CXRs). Few clinicians prefer daily routine CXRs in ICU patients, while others think that CXRs should be obtained on-demand when there is a reasonable clinical suspicion that an abnormality will be present [2–7].

 Although in the 1970s chest radiography was a part of routine tests performed in intensive care units, several studies have challenged its need [8–10]. Previous literature identified that chest radiography is a labor-intensive strategy and are moderately accurate in the diagnosis of pulmonary opacities [11] and lacks significant influence on clinical management for ICU’s patients [6, 13–15]. Moreover, they cause a heavy logistic and financial burden [4, 12, 13].

From a safe point of view, approximately 15% of nosocomial infections in the ICU result from the spread of bacteria on caregivers’ hand. The routine chest radiograph provides an unexamined opportunity for bacterial spread [16]. Patient’s exposure to unnecessary radiation is one of the main concerns in the argument against routinely performed chest radiographs for ICU patients [17, 18]. The radiation dose received by both the patients and healthcare workers during the procedure would be minimized by a reduction in the number of radiographs taken daily [11, 19, 20]. There is plenty of persuasive evidence which has been provided by results from several studies about the relatively minor benefits of routine chest radiograph in mechanically ventilated and intubated patients in the ICUs and the advantage of replacing the previous one with the on-demand chest radiograph protocol without compromising patient care and safety [2, 3, 10, 13, 14, 21–23].

The reasons given for the continuous use of the daily routine chest radiographs in the ICUs seems to be losing ground in the face of the numerous studies and clinical facts that keep emerging. Moreover, the on-demand protocol would provide the same value with better advantages in many areas where the daily routine CXR is found lacking [24].

A huge number of chest radiographs are ordered in medical centers across the United States annually. Similarly, it’s the same scenario in Saudi Arabia ICUs too. The overall aim of the research is to provide empirical evidence-base from ICUs clinicians’ perspective to inform policy and decision makers in Saudi Arabia about the daily routine chest radiograph in ICUs, in comparison with the on-demand portable chest radiograph; and to what extent the policy in place could be changed in favor of using on-demand instead of the current daily routine policy.

## Methods

This was a cross-sectional survey using a self-administered questionnaire. The target participants were the ICU physicians (Intensivists), ICU nurses and the respiratory therapists with primary assignment in ICU, who have worked in the ICU for more than six months. The study settings included all Intensive Care Units (ICU) of the tertiary hospitals in Saudi Arabia representing the main public, private, military and security forces. All these were referral hospitals, within total of 35 ICUs namely: National Guard Health Affairs- King Abdulaziz Medical City (9 ICUs), King Faisal Specialist Hospital and Research Center (4 ICUs), King Fahad Medical City (2 ICUs), King Saud medical city (3 ICUs), Security Forces Hospital (2 ICUs), King Abdulaziz University Hospital (2 ICUs), Armed Forces Hospital South Region, Khamis Mushayt (3 ICUs), Prince Sultan Military Medical City (3 ICUs), and Dr. Sulaiman Al-Habib Medical Group (3 ICUs), Dr. Abdulrahman Al Mishari Hospital (1 ICU), Dr. Barksh Hospital (1 ICU), Sultan Bin Abdulaziz Humanitarian City (2 ICUs).

### Procedure

The scientific and ethical approval of the study was granted by the research committee of the Public Health College and King Abdullah International Medical Research Center (KAIMRC) with IRB approval # SP14/044. Administrative permission was also obtained from the necessary authorities of all participating hospitals, and participants’ informed consent was attached with the study survey. Most of the questionnaires were hand-delivered to the majority of the hospitals in Riyadh, while the remaining questionnaires were sent via email to designated persons in hospitals at other regions in the Kingdom of Saudi Arabia. An introductory letter informing the participants about the researcher and the purpose of the study was also attached. The distribution started in the middle of June 2014 and the last batch of the questionnaire was received during the third week of October 2014. Self-administered questionnaires were distributed to most of the tertiary hospitals of Kingdom of Saudi Arabia (KSA). The first part was about demographic characteristics questions such as gender, age, nationality, qualifications, work experience and hospital/ICU-setting questions, and the second part dealt with clinical questions concerning chest radiography and reasons for doing the procedure. At the end of the second part of the questionnaire, participants were asked if they would recommend changing their hospital policy from daily routine to on-demand chest radiograph in a ‘Yes’ or ‘No’ format (see Additional file 1). Demographic characteristics were included in order to analyze the relationship between responders' demographic characteristics and their opinion about the radiograph procedure. All clinicians working in the cardiac and pediatric ICUs, and all other healthcare providers whose primary assignment areas were not in the ICU were excluded from this study.

### Data analysis

Data collected were entered into SPSS package version 20.0. Statistical analyses were done; descriptive and inferential analyses. The level of *p* ≤ 0.05 was used to indicate statistical significance. All variables were summarized and reported across the study subjects using descriptive statistics. The mean age of the participants was 35 ± 8 years. Categorical variables under (demographics, ICU characteristics, chest radiography data, and healthcare provider’s perception) were summarized and reported in terms of the frequency distribution (N (%). Chi-square / Fisher exact test was used to compare health care provider’s perceptions about on-demand chest radiography across categorical demographic characteristics. Results were reported in terms of count, percent, and *p*-value. For continuous variables (Age), normality assumption was first tested and verified using Kolmogorov-Smirnov test (*P*- < 0.0001), then independent sample T-test was used to compare between different perceptions, and the level of significance was set at *p* ≤ 0.05.

## Results

### Response rate and respondents/ ICU characteristics

Out of 730 questionnaires sent to study respondents, we received 495 completed questionnaires representing 67.8% response rate of the anticipated participants responded to the survey. Table 1 shows the respondents and ICU characteristics. About two-thirds (66.6%) of respondents were females, the Saudi nationality was 14.5% and the rest were from more than six international regions, especially the Asians and they accounted for 59.3% and the remaining 40.7% clinicians were collectively from the Middle East, Europe, Africa and North America. Only 16.9% of the participants had a diploma and the rest of the participants had a bachelor degree or higher. Nearly 38.9% of the respondents worked at the Ministry of National Guard Health Affairs in Riyadh while the remaining participants were from different public and private hospitals across regions in Saudi Arabia. One-third of the respondents (35.5%) have more than 10 years working experience in the ICU, 36.1% have been working in the ICU for between 6 and10 years while 28.4% have work experience in the ICU between 1 and5 years. The majority of the respondents (92.3%) have been working in the ICU for more than 6 months. More than two-thirds (70.9%) of the ICU Units belong to academic hospitals compared to 24% who were from non-academic hospitals. About half (49.9%) are open format ICU (i.e. other doctors apart from intensivists can make treatment orders for ICU patients) and 46.9% are close format (only intensivists can make treatment orders). More than half of the respondents (51.2%) have been working in a hospital setting with more than 20 ICUs beds and only 3.2% were from the hospital with less than 5 ICU beds, while the remaining hospitals had ICUs with beds ranging between 5 and 20.

**Table 1:** Respondents and ICUs Characteristics

Background characteristics	N (%)
Age (Means ± SD)	35 ± 8 years
Gender	
Male	160 (32.3)
Female	330 (66.6)
Missing	5 (1.1)
Nationality	
Saudi	72 (14.5)
Western	15 (3)
Middle Eastern	44 (8.9)
Asian	294 (59.3)
European	27 5.5)
African	19 (3.9)
Missing	24 (4.9)
Workplace	
Ministry of National Guard Health Affairs	193 (38.9)
King Faisal Specialist Hospital and Research Center	62 (12.5)
King Fahd Medical City	2 (0.5)
Prince Sultan Medical Military City	49 (9.9)
Security Forces Hospital	49 (9.9)
King Saud Medical City	25 (5.1)
Other	115 (23.2)
Education level	
Diploma	84 (16.9)
Bachelor	323 (65.2)
Master	33 (6.7)
PhD	37 (7.5)
MBBs	16 (3.2)
Missing	2 (0.5)
General Work Experience	
1–5 Years	141 (28.4)
6–10 Years	179 (36.1)
11–15 Years	89 (18)
> 15 Years	87 (17.5)
Work Experience in ICU Units	
Less than 6 months	30 (6.1)
More than 6 months	457 (92.3)
Missing	8 (1.6)
Previous employment/training	
Saudi Arabia	161 (32.5)
North America	27 (5.4)
Middle East	42 (8.5)
Asia	180 (36.3)
Europe	29 (5.8)
South African	11 (2.2)
Others	41 (8.3)
Missing	4 (1.0)
Current Position	
Consultant	32 (6.5)
Associate Consultant	5 (1.0)
Assistant Consultant	8 (1.6)
Staff Physician	19 (3.8)
Fellow	4 (0.8)
Resident	14 (2.8)
Respiratory Therapist	60 (12.1)
Nurse	353 (71.4)
ICU characteristics	N (%)
Number of ICU –beds available for mechanical ventilation	
< 5	16 (3.2)
5–15	116 (23.6)
16–20	107 (21.6)
> 20	254 (51.2)
Missing	2 (0.4)
Type of hospital	
Academic	351 (70.9)
Non-academic	119 (24.0)
Missing	25 (5.1)
Number of staff under training for intensivists	
None	97 (19.6)
1–4	185 (37.3)
> 4	158 (32)
Missing	56 (11.1)
Type of ICU	
Closed format	232 (46.9)
Open format	247 (49.9)
Missing	16 (3.2)

### Daily routine radiograph versus on-demand chest radiograph

**Fig. 1:**
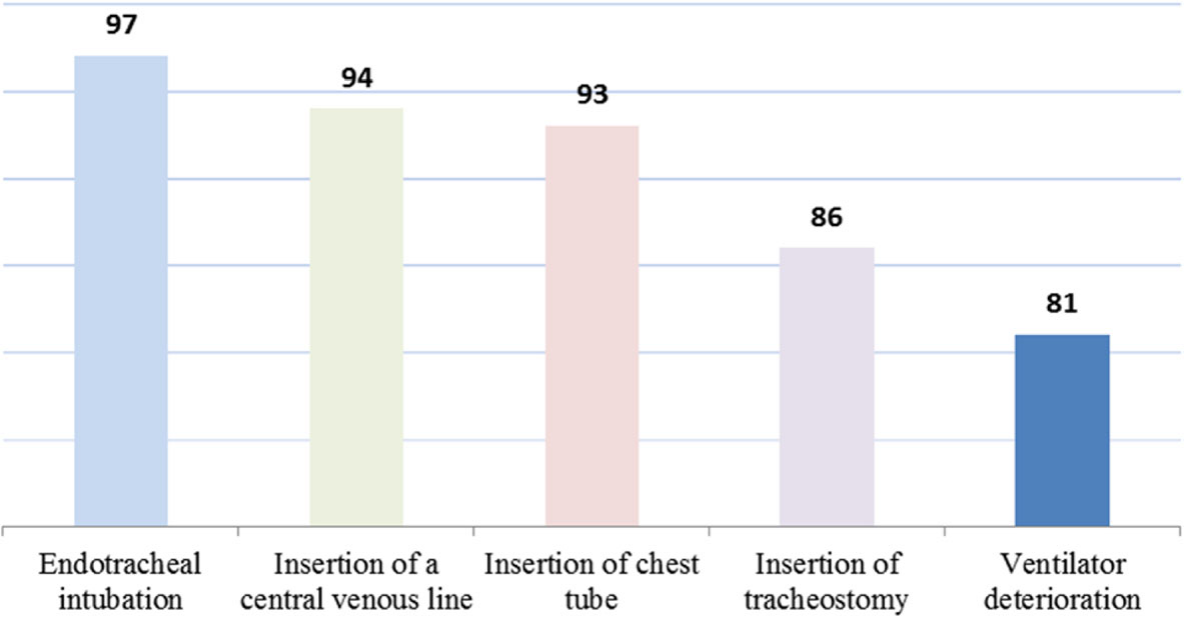
The Top Five Reasons for Daily chest radiography at ICUs (%)

The top five Reasons for daily chest radiography at ICUs in Saudi Arabia were endotracheal intubation, insertion of a central venous line, chest tube, and tracheostomy and in ventilator deterioration (Fig. 1). A daily routine chest radiograph was performed in almost 96.8% of the participants ICUs as a daily routine. More than two-thirds (64%) of clinicians reported that daily routine chest radiograph was performed in their ICU for all admitted patients and 19.4% of clinicians said it was for an only specific patient group in comparison to 33.3% clinicians performing only for intubated and mechanically ventilated patients [Table 2]. As shown in Table 3, about one-third (35.9%) of the participants agreed that daily routine CXRs influences patient care, while similar percentage (35.2%) of the participants agreed to the same rating for the on-demand policy and the remaining in the both categories thought that CXRs influence was less than 60% for the ICU patient care management. Interestingly, the results of the question concerning chest radiograph essentiality for the judgment of the presence or absence of Acute Respiratory Distress Syndrome (ARDS) shows that 93.3% of the clinicians agreed on the importance of chest radiograph in the early detection and treatment which is very crucial to optimize treatment outcomes; because patients with ARDS are at a very high risk for complications such as pneumonia (92.3%) and pneumothorax (93.5%). (Table 3).

**Table 2:** Daily routine chest x-ray practice

The daily routine chest X ray is commonly	N (%)
Performed in our ICU	
Yes	479 (96.8)
No	16 (3.2)
Performed in our ICU for all admitted patients	
Yes	317 (64)
No	178 (36)
Performed in our ICU, but only for intubated and mechanically ventilated patients	
Yes	165 (33.3)
No	330 (66.7)
Performed in our ICU, but only in specific patient group	
Yes	97 (19.6)
No	398 (80.4)
A Chest radiograph is always performed after	
Endotracheal intubation	
Yes	479 (96.8)
No	16 (3.2)
Insertion of a central venous line	
Yes	468 (94.5)
No	27 (5.5)
Insertion of a pulmonary artery catheter	
Yes	362 (73.0)
No	133 (27.0)
Insertion of tracheostomy	
Yes	428 (86.5)
No	67 (13.5)
Insertion of chest tube	
Yes	460 (92.9)
No	35 (7.1)
Ventilator deterioration	
Yes	400 (80.8)
No	95 (19.2)

**Table 3:** Clinicians perspective on the benefits of the chest radiography practice

Variable	N (%)
In your opinion, the daily routine chest radiograph influences care of patient in:	
< 10%	24 (4.8)
10–20%	53 (10.7)
20–30%	75 (15.1)
30–60%	151 (30.5)
> 60%	178 (36)
Missing	14 (2.9)
In your opinion, the on-demand chest radiograph influences care of patients in	
< 10%	21 (4.2)
10–20%	35 (7.1)
20–30%	84 (16.9)
30–60%	164 (33.1)
> 60%	174 (35.2)
Missing	17 (3.5)
A chest radiograph is essential for the judgment of the following:	
Presence or absence of Acute Respiratory Distress Syndrome (ARDS)	
Yes	462 (93.3)
No	33 (6.7)
Presence or absence of pneumonia	
Yes	457 (92.3)
No	38 (7.7)
Presence or absence of pneumothorax	
Yes	463 (93.5)
No	32 (6.5)
Pulmonary Edema	
Yes	434 (87.7)
No	61 (12.3)
Position of central venous line	
Yes	457 (92.3)
No	38 (7.7)
Position of chest tube	
Yes	462 (93.3)
No	33 (6.7)
Position of intra-aortic counter pulsing device	
Yes	371 (75.0)
No	124 (25.0)
Recommending On- Demand Chest Radiography	
Yes	362 (73.1)
No	119 (24.0)
Missing	14 (2.9)

### Recommendation to move from daily routine to on demand CXR

Table 3 also shows the health care provider standpoints towards changing to on-demand chest radiology instead of a routine chest x-ray. The majority (73.1%) of the surveyed clinicians agreed that the current daily routine chest radiograph policy for patients in the ICUs should be replaced by the on-demand chest radiograph policy compared to only 24% in favor of keeping the current daily routine policy. These findings showed a high percentage of support to replace the current policy across all the demographic characteristics in the study survey with no significant difference in the recommendation of on-demand chest radiography across subject’s demographic characteristics (*P*-value > 0.05). Table 4 shows the pattern of on-demand chest radiography practice in ICU. The majority of the clinicians (56.5%) disagreed that an on-demand chest radiograph is always performed after circulatory deterioration (Table 4).

**Table 4:** Pattern of On- Demand chest radiography practice in ICUs

A Chest radiograph is always performed after:	N (%)
Circulatory deterioration	
Yes	215 (43.5)
No	280 (56.5)
Cardiopulmonary resuscitation	
Yes	289 (58.4)
No	206 (41.6)
Arrival in ICU	
Yes	403 (81.5)
No	93 (18.5)
Before removal of endotracheal tube	
Yes	172 (34.7)
No	323 (65.3)
After removal of endotracheal tube	
Yes	164 (33.1)
No	331 (66.9)
Before ICU discharge	
Yes	80 (16.2)
No	415 (83.8)
Insertion of other invasive devices	
Yes	312 (63.1)
No	183 (36.9)
Before the early morning around by ICU team	
Yes	332 (67.1)
No	163 (32.9)
Removal of chest tube	
Yes	360 (72.8)
No	135 (27.2)

Results also revealed that 78.9% of physicians working in the ICUs, 71.4% residents and 50% fellows agreed that the current policy should be replaced. A similar percentage was obtained from the ICU nurses, 75% in support of abandoning the current policy and 25% believed that the current policy should be retained. More than three-fourths (80%) of the respiratory therapists supported replacing the current policy with the on-demand chest radiograph protocol and only 20% disagreed with protocol changing (Table 5).

**Table 5:** Differences in clinician’s opinion on moving to On-demand chest-radiography across the demographic and work-related characteristics

Variable	Change to On-Demand Chest Radiography Recommendation		*P*-value
	N (%)	N (%)	
	Yes	No	
Age	35 ± 8 Years	34 ± 8 Years	0.221
Gender			
Male	120 (75)	40 (25)	0.852
Female	249 (75.5)	78 (24.5)	
Nationality			
Saudi	62 (86.1)	10 (13.9)	0.316
Western	11 (73.3)	4 (26.7)	
Middle Eastern	34 (77.2)	10 (22.8)	
Asian	215 (73.1)	79 (26.9)	
European	22 (81.5)	5 (18.5)	
African	14 (73.6)	5 (26.4)	
Workplace			
Ministry of National Guard Health Affairs	148 (76.6)	45 (23.4)	0.463
King Faisal Specialist Hospital and Research Center	45 (72.5)	17 (27.5)	
King Fahd Medical City	1 (50.0)	1 (50.0)	
Prince Sultan Medical Military City	38 (77.6)	11 (22.4)	
Security Forces Hospital	37 (75.5)	12 (24.5)	
King Saud Medical City	23 (92)	2 (8)	
Other	80 (69.6)	35 (30.4)	
Education level			
Diploma	59 (70.2)	25 (29.8)	0.320
Bachelor	245 (76.0)	78(24.0)	
Master	28 (84.8)	5 (15.2)	
PhD	29 (78.4)	8 (21.6)	
MBBs	10 (62.5)	6 (37.5)	
Work Experience			
1–5 Years	104 (73.8)	37 (27.2)	0.861
6–10 Years	137 (76.5)	42 (23.5)	
11–15 Years	68 (76.4)	21 (23.6)	
> 15 Years	68 (78.1)	19 (21.9)	
Work Experience in ICU units			
Less than 6 months	26 (86.6)	4 (13.3)	0.253
More than 6 months	340 (74.3)	117 (25.7)	
Current Position			
Consultant	26 (81.2)	6 (18.8)	772
Associate Consultant	4 (80.0)	1 (20.0)	
Assistant Consultant	5 (62.5)	3 (37.5)	
Staff Physician	15 (78.9)	4 (21.1)	
Fellow	2 (50.0)	2 (50.0)	
Resident	10 (71.4)	4 (28.6)	
Respiratory Therapist	48 (80)	12 (20)	
Nurse	265 (75)	88 (25)	

## Discussion

To our knowledge, little research was done in Saudi Arabia to determine the efficacy of on-demand CXR and to abandon routinely done radiography in ICU adult patients. Hence the primary aim of this study was to seek and ascertain the opinion of clinicians working in the ICUs in Saudi Arabia, through a survey in a questionnaire form, on whether it is time to change the current daily routine chest radiograph for ICU patients to the on-demand policy. The health professionals such as nurses and others were included in the survey because they have been involved in the daily care of the ICU patients, to execute doctor’s orders and follow up with radiology department including daily CXR. Thus we included all the clinical staff to get a comprehensive perspective on the current practice of CXR and their opinions to move to on-demand strategy. The results obtained were similar in almost all the categories of the survey’s clinical questions.

Our results showed striking evidence that daily routine chest radiograph policy is what is currently being practiced in Saudi Arabia. This opinion is in line with the results of Graat et al. who did a survey to establish chest radiograph practice in Netherlands. It was found that large numbers of intensivists still practice a daily routine [19]. In our present study, majority of the participants (96.8%) agreed that a chest radiograph is always performed after endotracheal intubation, 94.5% for insertion of central venous line, 92.9% for insertion chest tube, 86.5% for insertion of tracheostomy, and 73% for insertion of a pulmonary artery catheter. Our findings were consistent with previous studies results that daily routine chest radiograph was mostly performed to ascertain the positioning of medical devices, like central venous line and chest tube in mechanically ventilated patients [6, 8, 15, 19, 25]. However, proper positioning and regular assessment of abnormalities caused by such medical devices are necessary to improve critical care management [26]. The majority of participants (81.5%) replied that chest radiograph was always performed when the patient arrived in their ICU, while 67.1% of respondents opinion was that chest radiograph was always performed even before the patient is shifted to ICU. Previous studies showed that routine chest radiograph has moderate accuracy in detecting lung opacities. And elimination of this routine procedure showed neither significant changes in the ICU management nor the patient’s length of stay [14].

The results from our study showed the rampant rate at which chest radiographs have been ordered and performed in the ICU, many of these procedures were unnecessary and might not lead to change in treatment management [3], and there was a consensus among respondents on the question whether daily routine and on-demand chest radiographs influences care of the ICU patients. About three-fifth (73%) of the current study participants agreed that the current daily routine chest radiograph policy for patients in the ICUs should be replaced by the on-demand chest radiograph policy, while only 24% support retaining the current daily routine policy. However, previous studies emphasized the benefits from routine chest radiograph in patients with HIV and ARDS [27]. Another similar study found that CXR obtained after the change in the patient’s medical condition yielded better results than did routine radiograph [28]. On the other hand, it is also important to consider potential risks associated with radiation exposure. A relative radiation level (RRL) dose estimate should be lowered to reduce the adverse health effect from unncessary radiation exposure [1].

Our study findings revealed that majority of the health care providers working in the ICUs agreed that the current policy of routine radiographs should be abandoned. According to European referral guidelines for imaging over utilization of radiological services creates a significant burden on the health care system and also increases the risk of radiation exposure [29].

There exists two schools of thought and the debate on daily routine CXR is not yet settled. Little research is available on Clinician’s perspective about the routine radiography in ICU while most of the studies focused on the outcome efficacy between a routine and restrictive CXR in different conditions [24, 26, 28, 30].

All the ICU clinicians participated in our survey have equal influence and role that would help in moving towards this new policy and practice. And as long as the daily routine chest radiograph has been in place for so long, it would require a lot of awareness, willingness and campaign to start the change that would eventually lead to the replacement of the current policy. “Old habits don’t die young” and quality of health services may not improve without change, even though not all changes may bring an improvement. There has been enough research carried out on this topic that supported replacing the daily routine chest radiograph with the restrictive on-demand protocol, but because of concern and fear of patient safety from some of healthcare providers, this most needed intervention for change has been slow in reaching the final stage and getting to the tables of decision makers that would make the final say and approval.

### Strengths and limitations

The selection of hospitals across the various regions in the Kingdom of Saudi Arabia and the good  percentage of response from the clinicians working in the Intensive care units were the main strengths of this study. On the other hand, our study has few limitations. Firstly, the majority of clinicians working in Saudi Arabia were expatriates from many different countries so the chest radiography protocol in practice in their home countries might have an influence on their perspective. Secondly, local factors related to how chest radiographs were ordered and obtained varies from different hospitals, and the level of technology in the medical imaging department was not the same across the board in Saudi Arabia. The obtained data in the current study were not validated against picture archiving and communication system. Moreover, we did not include the opinions of radiologists in the study, which is highly recommended to be surveyed in any future research .

## Conclusions

It seems there was a semi-consensus among study respondents including Physicians, Nurses, and RTs to replace the daily routine chest radiograph for ICUs patients. The support of these clinicians whose primary assignment is in the ICUs in hospitals from all regions in the Kingdom of Saudi Arabia was very encouraging, because the support unanimously cut across participant’s nationalities, qualifications, work experiences and profession. Nevertheless, the researchers believe the view of those who are not in support of changing the current practice should not be discarded. If needed, further studies should be conducted to alleviate the fear of ICU clinicians to bring them on board.

## Additional file

Additional file1: Questionnaire used in the study. Part one: Demographic Characteristics. Part two: Questions regarding chest radiography. (PDF 270 kb)

## Abbreviations

CXR: Chest X-Ray
ICU: Intensive care unit
