# Supplementary material for: Daily routine versus on-demand chest radiograph policy and practice in adult ICU patients- clinicians’ perspective
Source: BMC Med Imaging. 2018 Apr 3;18:4. doi: 10.1186/s12880-018-0248-6 (PMC5883277; doi:10.1186/s12880-018-0248-6)
Supplement: Supplementary file 1 — Questionnaire used in the study. Part one: Demographic Characteristics. Part two: Questions regarding chest radiography. (PDF 270 kb) [file 12880_2018_248_MOESM1_ESM.pdf]

King Saud bin Abdulaziz University for Health Sciences
College of Public Health & Health Informatics

Questionnaire Survey on

"Is It Time To Change the Current Hospital's Policy from Daily Routine Chest Radiographs To On-Demand Chest Radiographs in ICUs in Saudi Arabia?:

Healthcare Providers' Perspective

Researcher contact :

ABDULLAH AL SHAHRANI
TEL. NO. (01) 8011111 EXT. 11123
FAX NO. (01) 8011111 EXT. 11669
PAGER NO. 2028
MOBILE: 0505277253
Email: shahraniab@ngha.med.

Dear Respected Participant,

My name is Abdullah Al Shahrani; Master's student of Health Systems and Quality Management Program at the College of Public Health and Health Informatics King Saud Bin Abdulaziz University for Health Sciences. I am also a supervisor of the Diagnostic Imaging Section of Medical Imaging Department of King Abdulaziz Medical City, King Fahad National Guard Hospital, in Riyadh, Saudi Arabia.

Currently I am working on my research project with the title: **Is It Time To Change the Current Hospital's Policy from Daily Routine Chest Radiographs To On-Demand Chest Radiographs in ICUs in Saudi Arabia?:*Healthcare Providers' Perspective***

The attachment is questionnaire survey consisted of two main parts in which we would like to know your opinions about practicing of the **daily routine**chest x-ray versus **on-demand** chest x-ray imaging in the ICUs. Your answers and opinion on this issue are highly valuable, and will contribute positively in shaping the future of thechest radiography practice and informing the policy makers and practitioners in the ICUsin Saudi hospitals.

Thus, this this survey aims primarily to collect the scientific opinion and the perspective of the health care providers working in the ICUs, and to what extent if it is time to change the daily routine chest radiograph to on-demand chest radiograph in ICUs in Saudi Arabia.

Filling up the attached questionnaire will not take more than 10 minutes, with many thanks to you in advance for your valuable time and support.

□ **Agree to participate** □ **Not agree to participate**

Thank you

Part one: Demographic Characteristics:

1. **Sex :** □ Male □ Female

2. Age: ____

3. **Nationality:** _________________________________

4. **Work place:**

   ☐ Ministry of National Guard Health Affairs ☐Riyadh ☐Jeddah ☐Dammam ☐Alhasa☐Madina

   ☐ King Faisal Special Hospital and Research Center (FKSH&RC)

   ☐ King Fahad Medical City (KFMC)

   ☐ Prince Sultan Medical Military City (PSMMC)

   ☐ Dammam University Hospital

   ☐ Security Forces Hospital (SFH)

   ☐ King Saud Medical City (KSMC)

   ☐ Others: _________________________

5. **Qualifications:**

   ☐ Diploma

   ☐ Bachelor

   ☐ Master

   ☐ PhD

   ☐ MBBs

6. **Work Experience:**

   ☐1 – 5 years

   ☐ 6 to 10 years

   ☐ 11 to 15 years

   ☐> 15 years

7. **Work experience in an ICU units**

   ☐ Less than 6 months

   ☐ More than 6 months

8. Previous countries of Employment / Training:

☐ Saudi Arabia

☐ North America

☐ Middle East

☐ Asia

☐ Australia

☐ Europe

☐ South Africa

☐ Others

9. Current position:

☐ Consultant

☐ Associate Consultant

☐ Assistant Consultant

☐ Staff Physician

☐ Fellow

☐ Resident

☐ Respiratory Therapist

☐ Nurse

10. Number of ICU –beds available for mechanical ventilation:

☐< 5

☐ 5 - 15

☐ 16 – 20

☐> 20

11. Type of hospital:

☐ academic

☐non-academic

12. Number of fellows in training for intensivist:

☐ None

☐ 1 – 4

☐> 4

13. Type of ICU:

*☐ Closed format

*☐ Open format

***CLOSED ICU:** Only the intensivist can make order and decide on the line of treatment for the ICU patients.
***OPEN ICU:** Other Physicians apart from the intenvisists can also make orders and the line of treatment.

Part two: Questions regarding chest radiography

*Could you Please check any answer or option mentioned below that represent your opinion or current practice in the ICU you are working in**:***

1. **Daily-routine chest radiography: (CHECK ALL THAT APPLY)**
   - ☐ Not performed in our ICU
   - ☐ Performed in our ICU for all admitted patients
   - ☐ Performed in our ICU, but only for intubated and mechanically ventilated patients
   - ☐ Performed in our ICU, but only in specific patient groups (specify)……..

2. **A chest radiograph is always performed after: (CHECK ALL THAT APPLY)**
   - ☐ Endotracheal intubation
   - ☐ Insertion of a central venous line
   - ☐ Insertion of a pulmonary artery catheter
   - ☐ Insertion of tracheostomy
   - ☐ Insertion of chest tube
   - ☐ Ventilator deterioration
   - ☐ Circulatory deterioration
   - ☐ Cardiopulmonary resuscitation
   - ☐ Arrival in ICU
   - ☐ Before removal of endotracheal tube
   - ☐ After removal of endotracheal tube
   - ☐ Before ICU discharge
   - ☐ Insertion of other invasive devices
   - ☐ Before the early morning round by ICU team.
   - ☐ Removal of chest tube.

3. **In your opinion, the daily routine chest radiograph influences care of patient in:**
   - ☐< 10%
   - ☐10 – 20%
   - ☐ 20 – 30%
   - ☐ 30 – 60%
   - ☐> 60%

4. In your opinion, the on demand chest radiograph influences care of patients in:

□< 10%

□ 10 – 20%

□ 20 – 30%

□ 30 – 60%

□> 60%

5. In your opinion, a chest radiograph is essential for the judgment of: (CHECK ALL THAT APPLY)

□ Presence or absence of Acute Respiratory Distress Syndrome (ARDS)

□Presence or absence of pneumonia

□ Presence or absence of pneumothorax

□ Pulmonary Edema

□ Position of central venous line

□ Position of chest tube

□ Position of intra-aortic counter pulsing device

6. Do you recommend changing the Hospital's policy from the daily routine chest radiographs to On-demand chest radiographs?

□ Yes □No

If No , please specify why

……………………………………………………………………………………………

……………………………………………………………………………………………

……………………………………………………………………………………………

……………………………………………………………………………………………

……………………………………………………………………………………………
